# 

**Published:** 1876-12

**Authors:** 


VOL. VII. DECEMBER, 1876. No. 12.

### THE SOUTHERN MEDICAL RECORD

PUBLISHED MONTHLY AT

### ATLANTA, GEORGIA.

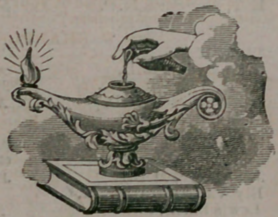

LOCAL EDITORS:

THOS. S. POWELL, M.D. R. C. WORD, M.D

W. T. GOLDSMITH, M.D. H. B. LEE, M.D.

ASSOCIATE EDITORS:

T. CURTIS SMITH, M.D........ MIDDLEPORT, OHIO.
SWAN M. BURNETT, M.D........ KNOXVILLE, TENN.
ALBAN S. PAYNE, M.D........... MARKHAM'S, VA.
A. R. KILPATRICK, M.D.......... NAVASOTA, TEXAS.
A. A. LYON, M.D.................. SHREVEPORT, LA.
G. A. BAXTER, M.D .............. CHATTANOOGA, TENN.
J. B. MATTISON, M.D............ BROOKLYN, N. Y.
E. T. EASLEY, A.M., M.D......... LITTLE ROCK, ARK.

"*QUICQUID PRÆCIPIES ESTO BREVIS.*"

ATLANTA, GA.:

JAS. P. HARRISON & CO., PRINTERS AND BINDERS.

1876.

